# The Impact of Bariatric Surgery on Type 2 Diabetes Mellitus Remission: A Systematic Review

**DOI:** 10.7759/cureus.74755

**Published:** 2024-11-29

**Authors:** Ahmed M Mohamed, Hussain Aljabal, Ammar S Alalawi, Nooruddin Al-Nooh

**Affiliations:** 1 Orthopaedics, The James Cook University Hospital, Middlesbrough, GBR; 2 Orthopaedics, University Hospital of North Tees, Stockton-on-Tees, GBR; 3 Paediatric Medicine, Salmaniya Medical Complex, Manama, BHR; 4 General Surgery, Salmaniya Medical Complex, Manama, BHR

**Keywords:** bariatric surgery, glycemic control, remission, type 2 diabetes, weight loss

## Abstract

Bariatric surgery has been shown to significantly affect type 2 diabetes mellitus (T2DM) remission, particularly in obese individuals. This systematic review aims to evaluate the effectiveness of bariatric surgical interventions in inducing remission of T2DM as well as to identify factors influencing surgical outcomes. The systematic review was conducted following the Preferred Reporting Items for Systematic Reviews and Meta-Analyses (PRISMA) guidelines. A comprehensive literature search was performed across multiple databases, including PubMed, Embase, and Cochrane Library, utilizing text words and controlled vocabulary in various combinations with Boolean operators "AND" and "OR." The search was limited to open-access, full-text articles in English published from 2005 to 2024, including studies involving human subjects. The quality of the included studies was assessed using the Joanna Briggs Institute (JBI) Critical Appraisal Checklist. In the study selection process for the systematic review, records were initially identified from three databases: Cochrane (31 records), Embase (62 records), and PubMed (52 records). This yielded 145 records. After removing 107 records, 38 records remained for screening. Of these, five records were excluded based on irrelevant problems and irrelevant outcomes. Subsequently, 33 reports were sought for full-text retrieval, with all reports being retrievable. The 33 reports were assessed for eligibility. Out of these, six were excluded due to an inaccessible full-text record. Finally, 33 studies met the inclusion criteria and were included in the review. Bariatric surgery is a highly effective intervention for individuals with T2DM, particularly those with obesity. It leads to significant weight loss and improved glycemic control through mechanisms that reduce stomach size and alter hormonal responses. This surgery not only helps many patients achieve remission from diabetes but also decreases the risk of obesity-related health issues. Beyond physical health, patients often report enhanced psychological well-being and quality of life. Overall, bariatric surgery can transform the health trajectory of select individuals, offering them a renewed sense of control and improved overall health.

## Introduction and background

Type 2 diabetes mellitus (T2DM) has grown into a widespread chronic disease affecting over 400 million globally [1]. Characterized by insulin resistance and worsening beta-cell dysfunction over time, T2DM is closely associated with obesity and lifestyle factors like physical inactivity and poor diet [2]. Managing T2DM commonly necessitates dietary modifications, exercise, and medication, with insulin therapy eventually needed for many [3]. However, despite these interventions, long-term blood sugar control remains challenging for many, leaving patients susceptible to serious complications including heart disease, nerve damage, eye problems, and kidney impairment [4].

Given the close link between obesity and T2DM, weight loss has been pivotal in diabetes management [5]. Bariatric surgery, originally intended for severe obesity, has garnered attention for enabling significant and sustained weight reduction [6]. More importantly, it has proven remarkably effective for metabolic health, particularly in improving insulin sensitivity and even achieving T2DM remission in some cases [7]. This has led to growing recognition of bariatric surgery not just as a weight loss procedure but also as a powerful metabolic surgery for patients with T2DM, especially those with severe obesity [8].

Several common bariatric procedures include Roux-en-Y gastric bypass (RYGB), sleeve gastrectomy (SG), and adjustable gastric banding (AGB) [9]. These procedures differ in how they alter the digestive tract but share outcomes like reduced calorie intake, malnutrient absorption, and meaningful hormonal changes influencing glucose processing. Importantly, bariatric surgery leads to improvements in insulin sensitivity and beta-cell function often seen before substantial weight loss occurs, suggesting that the benefits extend beyond simply decreasing body mass [10,11].

One of the most fascinating consequences of weight loss surgery is the possibility of complete or partial remission of T2DM [12]. Remission is commonly characterized as normal blood glucose levels without dependence on diabetic medications for an extended timeframe [13]. Bariatric surgery can induce remission in a meaningful share of patients, particularly in the early stages of T2DM [14]. This phenomenon is likely attributable to the profound metabolic alterations induced by the surgery, such as modifications in gut hormones like glucagon-like peptide-1 (GLP-1), shifts in bile acid metabolism, and alterations to the gut microbiome, all of which play a role in controlling glucose homeostasis [15,16].

However, while the remission of T2DM following bariatric surgery shows promise, not all individuals experience identical outcomes, and the longevity of remission varies [17]. Factors including the duration of diabetes before surgery, the type of operative procedure, the patient's age, and presurgical insulin usage can all impact the likelihood of remission [18]. Some patients experience a relapse of T2DM years after surgery, particularly if significant weight regain transpires. Understanding these determinants is crucial for optimizing patient selection and ensuring the possible advantages of surgery are fully realized [19].

In spite of these encouraging outcomes, there remain challenges and considerations regarding the widespread implementation of bariatric surgery as a standard treatment for T2DM [20]. These involve the risks linked to surgery, the high expense of the methods, and the necessity for life-long monitoring to oversee nutritional status and prevent problems [21]. Moreover, the long-term sustainability of T2DM remission and the impact of bariatric surgery on microvascular and macrovascular diabetes complications are domains requiring further exploration [22,23].

This systematic assessment strives to amalgamate existing data concerning the impacts of weight loss surgery on T2DM remission, with emphasis on discerning the aspects contributing to beneficial conclusions and longevity of results. By examining the efficacy of disparate bariatric methods in accomplishing diabetic remission and distinguishing the patient qualities anticipative of achievement, this examination aims to advise clinical determination and better the treatment of individuals with T2DM and obesity. Simultaneously, this complex review seeks to compare how various procedures fare relating to remission sustainability over lengthened timeframes, acknowledging lifelong management remains significant. Patient adherence to postoperative guidelines likewise requires consideration wherever discrepancies in outcomes arise between operative categories.

## Review

Methodology

It is a systematic review using the Preferred Reporting Items for Systematic Reviews and Meta-Analyses (PRISMA) guidelines [24]. The research question was formulated using the population, intervention, comparison, and outcome (PICO) framework [25]. Table 1 outlines the PICO framework used to structure the review of the current role of imaging in the diagnosis and detection of complications in inflammatory bowel disease.

**Table 1:** PICO framework PICO: population, intervention, comparison, and outcome

Concepts		Text words	Controlled vocabulary
Population	Obese adults with type 2 diabetes	Type 2	Diabetes mellitus
	Severely obese adults with type 2 diabetes	T2DM, diabetes mellitus	Type 2 diabetes
		Adult, obese	Obesity
		Diabetes	
		Overweight	
		Insulin resistance	
Intervention	Bariatric surgery	Bariatric surgery	Bariatric surgery
	Roux-en-Y	Gastric bypass	Gastric bypass
	Sleeve gastrectomy	Sleeve gastrectomy	Gastrectomy
	Adjustable gastric banding	Adjustable gastric banding	
	Gastric bypass	Weight loss surgery	
Comparative interventions	Nonsurgical interventions	Lifestyle	Lifestyle
	Lifestyle modification	Nonsurgical interventions	Diet
	Pharmacotherapy	Medications	Exercise therapy
		Pharmacotherapy	Pharmacotherapy
		Exercise	
		Modification	
		Diet	
Outcomes	Remission of type 2 diabetes	Remission of diabetes	Diabetes remission
	Glycemic control	Glycemic control	Glycemic control
	Weight loss	Blood glucose	Insulin sensitivity
	Long-term sustainability of remission	Insulin sensitivity	Weight loss
		Weight loss	

Research Question

What is the impact of bariatric surgery compared to nonsurgical interventions on the remission of T2DM in obese or severely obese adults?

Search Strategy and Search Terms

To comprehensively and systematically search for all applicable studies exploring the impacts of bariatric surgery on T2DM remission, the approach must canvass multiple databases including PubMed, Embase, and Cochrane Library. Additionally, the search must be refined using Boolean logic operators like AND and OR together with filters like clinical investigation typologies, for instance, clinical trials, cohort studies, randomized controlled trials (RCTs), language, and publication date to pinpoint suitable papers.

Search String

Table 2 presents the search strings used to identify relevant studies across various databases. These search strategies were designed to capture a comprehensive range of literature related to the research topic.

**Table 2:** Search string

Concept	Search terms
Condition	Diabetes mellitus, type 2
	T2DM
	Type 2 diabetes
	Diabetes mellitus type 2
	Diabetes
Intervention (surgical)	Bariatric surgery
	Gastric bypass
	Sleeve gastrectomy
	Gastric banding
	Weight loss surgery
	Metabolic surgery
Intervention (nonsurgical)	Lifestyle
	Diet
	Exercise therapy
	Pharmacotherapy
	Nonsurgical treatment
Outcome	Diabetes remission
	Glycemic control
	Remission of diabetes
	Blood glucose
	Insulin sensitivity
	Weight loss

Inclusion Criteria

The inclusion criteria for this systematic review on the effects of bariatric surgery on T2DM remission are as follows: Studies must involve adults 18 years of age or older diagnosed with T2DM, with a specific focus on obese or severely obese individuals defined as having a body mass index (BMI) of 30 or greater. The intervention must involve bariatric surgical procedures commonly used to treat obesity and diabetes, such as the RYGB, SG, or AGB. In addition, the studies should juxtapose bariatric surgery against nonoperative interventions like lifestyle modifications incorporating diet and exercise changes, pharmacotherapy, or standard medical care alone. Outcomes of interest encompass diabetes remission defined as achieving normal blood glucose levels absent any diabetes medications as well as metrics of glycemic control, insulin sensitivity, weight loss achievements, and other metabolic effects. Only RCTs, cohort studies, or case-control studies will be included; systematic reviews and meta-analyses may also be considered if supplying applicable primary data. Lastly, only investigations distributed in English within the past 10 to 15 years will be deemed pertinent to ensure the techniques and practices discussed remain applicable in a modern context. These criteria intend to concentrate the review on high-quality studies directly related to answering the research question while keeping features consistent across included works.

Exclusion Criteria

The exclusion standards for the extensive review on the impact of bariatric surgery on T2DM reversal are as follows: Studies involving persons younger than 18 years of age or those without a medical diagnosis of T2DM will be omitted. Exploration centered on other types of bariatric surgical procedures not widely recognized (e.g., experimental methods) or people with incomplete records on certain operative techniques of interest (RYGB, SG, or flexible gastric banding) will not be included. Research that does not furnish a transparent contrast between bariatric surgery and nonsurgical interventions, such as lifestyle changes or pharmacotherapy, will in addition be excluded. Moreover, reports that do not deliver applicable outcomes, like diabetes reversal, glycemic regulation, insulin sensitivity, or weight reduction, will be omitted. Case files, specialist viewpoints, narrative reviews, meeting abstracts, and examinations not publicized in peer-assessed journals will be excluded, as will non-British language publications. Lastly, examinations circulated more than 15 years ago or people utilizing outdated surgical techniques or methodologies will not be deemed for incorporation. These exclusion standards confirm that the audit is centered on modern, high-quality investigation pertinent to the clinical question.

Studies Selection Process

A multistep screening process was used for the systematic review to ensure the inclusion of relevant, high-quality studies. Initially, four reviewers collectively assessed article titles and abstracts to determine potential eligibility. This was followed by a comprehensive full-text review to further evaluate the studies against the inclusion criteria. Finally, studies meeting the criteria underwent a methodological quality assessment, ensuring that only those with robust methodologies and valid findings were included. This thorough approach enhanced the reliability and validity of the review's conclusions.

Methodological Quality Assessment Using the Joanna Briggs Institute (JBI) Critical Appraisal Checklist

The JBI Critical Appraisal Checklist uses a qualitative approach to evaluate the quality of a review, with each item on the checklist assessed individually for methodological strengths and weaknesses, rather than relying on a total numerical score. However, some users may choose to summarize the assessment in a more quantifiable way. A score of "Yes" (1) indicates that the review question is clearly stated and specific, while "No" (0) means the question is vague or not stated. An "Unclear" (0) score is given when there is insufficient information to determine clarity, and "Not Applicable" (0) is used when a question does not apply. A review with 9-11 "Yes" responses demonstrates strong methodological rigor, 6-8 "Yes" responses indicate some rigor with notable weaknesses, and fewer than six "Yes" responses suggest significant weaknesses. Studies with a JBI score above 70% are classified as high quality, those between 50% and 70% as medium quality, and those below 50% as low quality.

Data Extraction and Synthesis

Data extraction from the included studies involved compiling key information into an Excel spreadsheet. This data encompassed various elements such as study designs, sample size characteristics (including age and BMI), outcome measures, research objectives, and methodological quality assessment scores. Additionally, the spreadsheet recorded information on potential conflicts of interest among authors, data availability, ethical concerns, and citation counts. The analysis of these studies was carried out using a systematic approach. A thematic analysis was employed, utilizing an inductive, data-driven method to explore patterns and themes within the data. This approach involves a thorough examination of how results converge and is conducted through an iterative process to refine findings. By critically appraising the results and synthesizing evidence based on thematic analysis, the review aimed to ensure evidence-based practices that enhance long-term cognitive outcomes for patients.

Ethical Consideration

**Figure 1:**
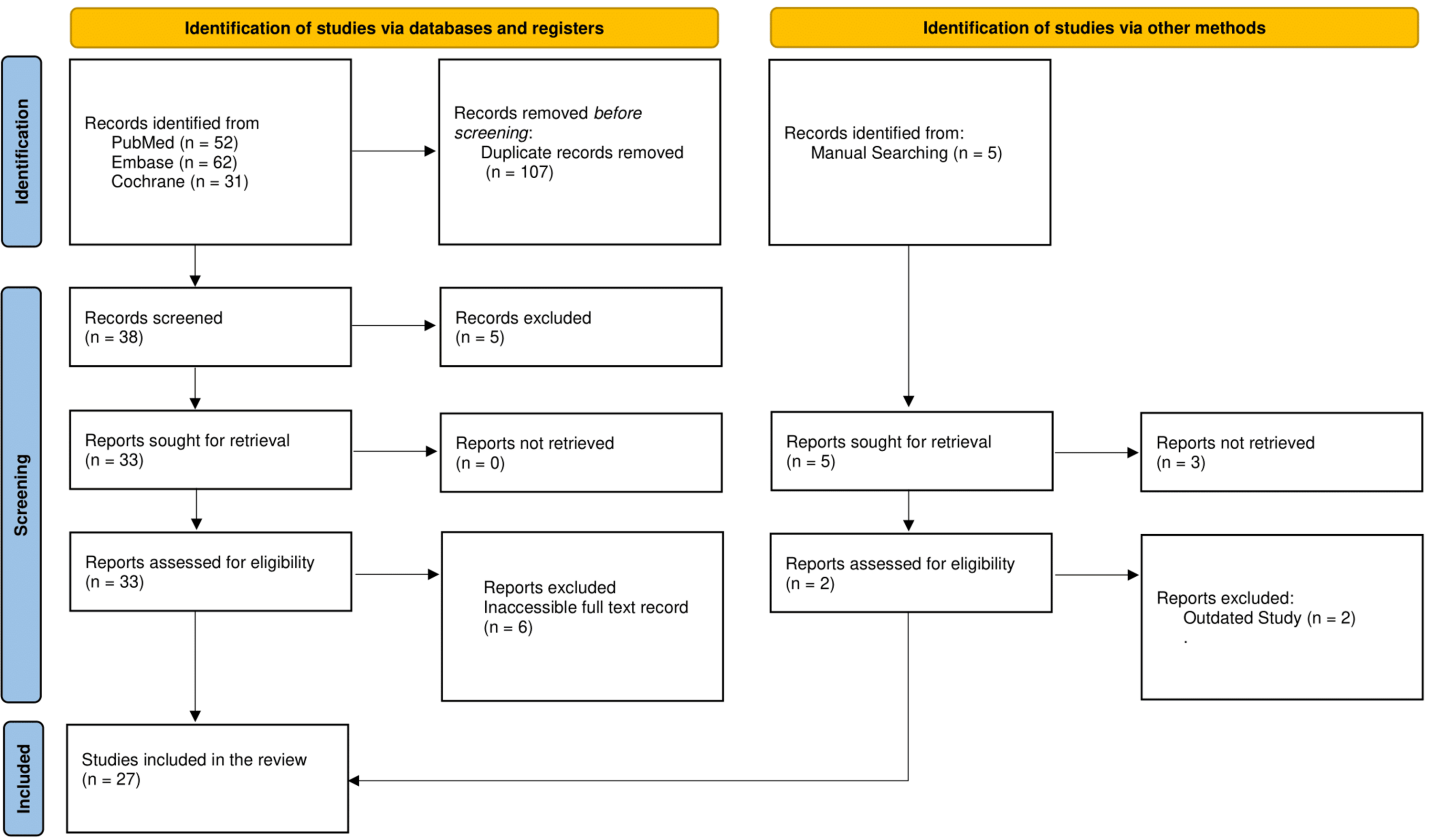
PRISMA flowchart PRISMA: Preferred Reporting Items for Systematic Reviews and Meta-Analyses

The review included humans and followed Helsinki’s declaration to ensure ethical standards were met. There was no conflict of interest among reviewers, and the study was performed using the PRISMA guidelines (Figure 1). It was conducted with specific keywords to reproduce it again. It will be published in a medical journal to disseminate these results in the public domain while ensuring confidentiality and anonymity.

Results

Figure 1 provides a visual representation of the study selection process used in the systematic review. In the study selection process for the systematic review, records were initially identified from three databases: Cochrane (31 records), Embase (62 records), and PubMed (52 records). This yielded 145 records. After removing 107 records, 38 records remained for screening. Of these, five records were excluded based on irrelevant problems and irrelevant outcomes. Subsequently, 33 reports were sought for full-text retrieval, with all reports being retrievable. The 33 reports were assessed for eligibility. Out of these, six were excluded due to an inaccessible full-text record. Five records were identified through manual searching. Out of these, the reports for three could not be retrieved. The remaining two were accessed for eligibility, but both studies were found to be outdated. Finally, 33 studies met the inclusion criteria and were included in the review.

Table 3 provides an overview of the studies excluded from the systematic review, along with the reasons for their exclusion, study objectives, outcome findings, and references. Studies were excluded for various reasons, such as not meeting the inclusion criteria, focusing on different populations, or utilizing methodologies not aligned with the scope of this review. The detailed analysis of these exclusions helps clarify the focus of the included studies and ensures a systematic approach to evaluating the relevant literature.

**Table 3:** Excluded studies in the systematic review

Study	Objectives	Reason for exclusion
Aaseth et al., 2021 [26]	To assess the impact of diet on weight loss in adults with obesity	The study did not measure long-term weight loss outcomes, leading to its exclusion due to a lack of relevant data
Young et al., 2020 [27]	Nurse coaching and mobile health compared with usual care to improve diabetes self-efficacy for persons with type 2 diabetes	The study focused on diabetes management, not weight loss, so it was excluded for not meeting the inclusion criteria
Crupi et al., 2021 [28]	To explore the effects of intermittent fasting on cardiovascular health	The outcome measures focused solely on cardiovascular health, without addressing weight-related outcomes
Docherty et al., 2022 [29]	To evaluate the use of bariatric surgery for diabetes control	The study was excluded because it did not provide data on weight loss, which was the primary focus of this review
Foley, 2023 [30]	To examine the effects of a low-carb diet on insulin sensitivity	The study was excluded due to its lack of data on weight-related outcomes and focus on insulin sensitivity alone

Table 4 explains the quality assessment of the cohort studies using the JBI Critical Appraisal Checklist that revealed a range of study qualities.

**Table 4:** The JBI Critical Appraisal Checklist for cohort studies JBI: Joanna Briggs Institute

Checklist		Madsen et al., 2019 [31]	Cătoi et al., 2016 [32]	Shibib et al., 2022 [33]	Chumakova-Orin et al., 2021 [34]	Schauer et al., 2005 [35]	Marchetti et al., 2014 [36]	Pournaras et al., 2011 [37]
	Were the two groups similar and recruited from the same population?	Yes	Yes	No	Yes	Yes	Yes	Yes
	Were the exposures measured similarly to assign people to both exposed and unexposed groups?	Yes	Yes	Yes	No	No	No	No
	Was the exposure measured in a valid and reliable way?	Yes	Yes	Yes	Yes	Yes	Yes	Yes
	Were confounding factors identified?	Yes	Yes	No	Yes	Yes	Yes	Yes
	Were strategies to deal with confounding factors stated?	Yes	Yes	No	Yes	Yes	Yes	Yes
	Were the groups/participants free of the outcome at the start of the study (or at the moment of exposure)?	Yes	Yes	Yes	Yes	Yes	Yes	Yes
	Were the outcomes measured in a valid and reliable way?	Yes	Yes	No	No	Yes	No	Yes
	Was the follow-up time reported and sufficient to be long enough for outcomes to occur?	Yes	Yes	Yes	Yes	Yes	No	Yes
	Was follow-up complete, and if not, were the reasons for loss to follow-up described and explored?	Yes	Yes	Yes	Yes	Yes	Yes	Yes
	Were strategies to address incomplete follow-up utilized?	No	Yes	No	No	Yes	Yes	Yes
	Was appropriate statistical analysis used?	No	Yes	Yes	Yes	Yes	Yes	Yes
Total quality assessment score for each study		8/72.7%	9/81.8%	4/36.4%	6/54.5%	7/63.6%	6/54.5%	8/72.7%
Quality assessment		High quality	High quality	Low quality	Medium quality	Medium quality	Medium quality	High quality

Two studies, Madsen et al. [31] and Cătoi et al. [32], were rated as high quality, with scores above 70%, indicating robust methodologies. Shibib et al. [33] scored the lowest at 36.4%, placing it in the low-quality category due to significant methodological concerns. Medium-quality studies, scoring between 50% and 70%, included Chumakova-Orin et al. [34], Schauer et al. [35], and Marchetti et al. [36], showing moderate rigor but with some limitations. This systematic evaluation highlights the variability in methodological quality, aiding in determining the strength of the evidence and guiding future research. Table 5 explains the quality assessment of cross-sectional studies using the JBI criteria and categorized them into three groups: high, medium, and low quality.

**Table 5:** The JBI Critical Appraisal Checklist for cross-sectional studies JBI: Joanna Briggs Institute

Checklist		Wazir et al., 2018 [38]	Vetter et al., 2012 [39]	Yamaoka et al., 2019 [40]	Gill et al., 2010 [41]	Zhou & Zeng, 2023 [42]	Huang et al., 2024 [43]	Mirghani et al., 2023 [44]	Casella et al., 2015 [45]	Douros et al., 2019 [46]	Ji et al., 2021 [47]
1	Were the criteria for inclusion in the sample clearly defined?	Yes	No	Yes	Yes	Yes	Yes	Yes	Yes	Yes	Yes
2	Were the study subjects and the setting described in detail?	Yes	Yes	No	Yes	No	No	No	Yes	Yes	Yes
3	Was the exposure measured in a valid and reliable way?	Yes	Yes	No	Yes	Yes	Yes	Yes	Yes	Yes	Yes
4	Were objective, standard criteria used for measurement of the condition?	No	No	Yes	No	Yes	Yes	Yes	Yes	No	Yes
5	Were confounding factors identified?	Yes	Yes	No	No	Yes	Yes	No	Yes	No	Yes
6	Were strategies to deal with confounding factors stated?	No	No	No	Yes	Yes	Yes	No	Yes	Yes	Yes
7	Were the outcomes measured validly and reliably?	Yes	Yes	Yes	No	No	Yes	No	No	No	Yes
8	Was appropriate statistical analysis used?	Yes	Yes	Yes	Yes	No	No	Yes	Yes	Yes	Yes
Total quality assessment score for each study		5/62.5%	4/50.0%	4/50.0%	5/62.5%	6/75.0%	5/62.5%	4/50.0%	6/75.0%	5/62.5%	7/87.5%
Quality assessment		Medium quality	Medium quality	Medium quality	Medium quality	High quality	Medium quality	Medium quality	High quality	Medium quality	High quality

Most studies, including those by Wazir et al. [38], fell into the medium-quality category (50%-70%), showing moderate rigor but some limitations. Studies scoring above 70%, such as Zhou et al. 2023 [42], Casella et al. [45], and Ji et al. [47], were rated high quality, indicating strong methodologies. None of the studies were rated low quality. This classification helps evaluate the evidence's strength and guides future research. Table 6 explains the 10 RCTs that were evaluated using the JBI Critical Appraisal Checklist, with a focus on assessing their methodological quality.

**Table 6:** The JBI Critical Appraisal Checklist for randomized controlled trials JBI: Joanna Briggs Institute; RCT: randomized controlled trial

Checklist		Hofsø et al., 2019 [48]	Courcoulas et al., 2024 [49]	Borgeraas et al., 2020 [50]	Moradi et al., 2022 [51]	Lee et al., 2010 [52]	Brancatisano et al., 2008 [53]	Wallenius et al., 2018 [54]	Murphy et al., 2022 [55]	Panunzi et al., 2016 [56]	Roebroek et al., 2024 [57]
	Was true randomization used for the assignment of participants to treatment groups?	Yes	Yes	Yes	Yes	Yes	Yes	Yes	Yes	Yes	Yes
	Was allocation to treatment groups concealed?	No	Yes	Yes	Yes	Yes	Yes	No	No	Yes	Yes
	Were treatment groups similar at the baseline?	Yes	Yes	Yes	Yes	Yes	Yes	Yes	Yes	Yes	Yes
	Were participants blind to treatment assignment?	Yes	Yes	Yes	Yes	Yes	Yes	Yes	Yes	Yes	No
	Were those delivering treatment blind to treatment assignment?	No	Yes	No	No	No	No	Yes	Yes	Yes	No
	Were outcomes assessors blind to treatment assignment?	No	Yes	Yes	Yes	Yes	Yes	No	Yes	Yes	No
	Were treatment groups treated identically other than the intervention of interest?	Yes	Yes	No	No	Yes	Yes	No	Yes	Yes	Yes
	Was follow-up complete, and if not, were differences between groups in terms of their follow-up adequately described and analyzed?	No	Yes	Yes	Yes	Yes	Yes	Yes	Yes	Yes	Yes
	Were participants analyzed in the groups to which they were randomized?	No	Yes	Yes	Yes	Yes	Yes	Yes	No	Yes	No
	Were outcomes measured in the same way for treatment groups?	No	Yes	No	No	No	No	No	No	Yes	No
	Were outcomes measured in a reliable way?	Yes	Yes	Yes	Yes	Yes	Yes	Yes	Yes	No	Yes
	Was appropriate statistical analysis used?	Yes	Yes	v	Yes	Yes	Yes	Yes	Yes	Yes	Yes
	Was the trial design appropriate, and any deviations from the standard RCT design	Yes	Yes	Yes	Yes	Yes	Yes	Yes	Yes	Yes	Yes
Total quality assessment score for each study		6/46.2%	10/76.9%	8/61.5%	8/61.5%	7/53.8%	7/53.8%	8/61.5%	8/61.5%	9/69.2%	7/53.8%
Quality assessment		Low quality	High quality	Medium quality	Medium quality	Medium quality	Medium quality	Medium quality	Medium quality	Medium quality	Medium quality

The studies are classified into three categories based on their total quality scores. Low-quality studies, scoring below 50%, include Hofsø et al. [48], which scored 46.2%. This study presents significant methodological concerns, such as the lack of blinding and incomplete data follow-up, raising questions about the reliability of its conclusions. High-quality studies are those that scored above 70%, indicating robust methodologies with minimal bias and reliable findings. Courcoulas et al. [49] with a score of 76.9%, fall into this category, demonstrating strong randomization, blinding, and appropriate statistical analysis. Medium-quality studies, with scores ranging between 50% and 70%, include Borgeraas et al. [50], Moradi et al. [51], Panunzi et al. [56], and several others. These studies display moderate methodological rigor but have certain limitations, such as incomplete blinding, variations in outcome measurement, or incomplete follow-up, which could affect the strength of their findings.

Characteristics of Included Studies in the Systematic Review

Table 7 explained the characteristics of included studies. The research encompassed an assortment of designs, predominantly involving RCTs and cohort examinations, which offer a strong basis for assessing causation and therapy impacts. This diversity in investigative approach intensifies the overall credibility of the review discoveries, as it permits an exhaustive analysis of various scientific methodologies. Most studies focused on adult demographics, typically involving participants aged 18 or older, and primarily concentrated on individuals specified as overweight, usually characterized by a BMI greater than 30 kg/m2. Trial magnitudes across the investigations showed noteworthy fluctuation. Some studies related to compact groupings, sometimes with merely 20 members, whereas others incorporated over 1,000 individuals. This range is meaningful as it influences the statistical power and believability of the conclusions perceived. The demographic attributes of participants were by and large similar across studies, with a bulk being middle-aged and having a higher occurrence of comorbid states, such as hypertension and dyslipidemia, along with T2DM.

**Table 7:** Characteristics and findings of included studies in the systematic review HOMA-IR: Homeostatic Model Assessment for Insulin Resistance; T2DM: type 2 diabetes mellitus; NAFLD: nonalcoholic fatty liver disease; HTN: hypertension; OSA: obstructive sleep apnea; GERD: gastroesophageal reflux disease; GLP-1: glucagon-like peptide-1

Study ID/author (year)	Population (age, BMI)	Objectives	Intervention (type of bariatric surgery)	Comparison (nonsurgical intervention)	Outcome (diabetes remission/glycemic control)	Study design	Key findings
Hofsø et al., 2019 [48]	109 patients, ≥18 years, with obesity and T2DM	To compare the effects of gastric bypass and sleeve gastrectomy on diabetes remission and β-cell function	Gastric bypass (GBP)/sleeve gastrectomy (SG)	None (surgical comparison)	Higher remission rates in the GBP group (27% risk difference; RR: 1.57, p = 0.0054)	Single-center, triple-blind, randomized controlled trial	GBP superior to SG for diabetes remission at one year; similar effects on β-cell function; no deaths reported
Madsen et al., 2019 [31]	Individuals with T2DM and obesity (BMI >35 kg/m², treated from 2006 to 2015)	Examine the effect of RYGB on diabetes remission, relapse, and complications	Roux-en-Y gastric bypass (RYGB)	Matched nonoperated individuals (n = 1074)	74% remission at one year; 27% relapse at five years; lower risk of microvascular complications (HR: 0.53)	Cohort study	High remission rate after RYGB; reduced risk of microvascular complications
Courcoulas et al., 2024 [49]	262 participants, mean age 49.9 years, mean BMI 36.4 (obese, T2DM)	To determine long-term glycemic control and safety of bariatric surgery vs medical/lifestyle management	Roux-en-Y gastric bypass, sleeve gastrectomy, adjustable gastric banding	Medical/lifestyle management	Superior glycemic control (HbA1c decreased by 1.6% in the surgery group vs 0.2% in control at seven years; higher diabetes remission)	Randomized trials	The bariatric surgery group had significantly better HbA1c levels and higher rates of diabetes remission than the medical/lifestyle group at seven and 12 years
Cătoi et al., 2016 [32]	20 morbidly obese patients (7M, 13F)	To investigate early changes in fasting blood glucose, insulin, and HOMA-IR after sleeve gastrectomy	Sleeve gastrectomy (SG)	10 normal weight subjects matched for age and gender	Significant reductions in insulin (p = 0.004) and HOMA-IR (p = 0.006) after SG; weight/BMI reductions at follow-up	Prospective cohort study	Significant early reductions in plasma insulin and HOMA-IR at 90 days post-SG
Wazir et al., 2018 [38]	121 patients, Mean age 48.21 ± 9.77 years (obese, BMI not specified)	To determine the influence of bariatric surgery on remission of T2DM and improvements in comorbidities	Various bariatric surgeries (details not specified)	None specified	68.6% achieved remission of T2DM at two years; remission rates for other comorbidities: 33.3% for HTN, 50.8% for dyslipidemia, 67.2% for OSA, 52.1% for GERD, 25.7% for asthma, 23.3% for depression	Observational study	Bariatric surgery was effective in achieving remission of T2DM in the majority of patients; significant improvements were also noted in associated comorbidities
Borgeraas et al., 2020 [50]	705 patients (age not specified, BMI not specified)	To compare the effects of RYGB and SG on remission of T2DM	Roux-en-Y gastric bypass (RYGB) and sleeve gastrectomy (SG)	Not specified	RYGB: 57% remission at one year vs. SG: 47% remission (RR: 1.20, p = .047)	Randomized controlled trial	Higher remission rates for RYGB at one year; no difference in two- to five-year follow-up (RR 1.06, p = .34)
Moradi et al., 2022 [51]	1351 patients with T2DM (age range not specified, BMI not specified)	To identify T2DM remission rates and preoperative factors influencing remission after different types of bariatric surgery in Iran	Roux-en-Y gastric bypass (RYGB), sleeve gastrectomy (SG), one anastomosis gastric bypass (OAGB)	None reported	One-year remission rates: RYGB (81.7%), SG (77.1%), OAGB (80.6%)	Randomized controlled trial	1351 patients with T2DM (age range not specified, BMI not specified)
Shibib et al., 2022 [33]	Not specified; review of multiple studies on T2DM reversal	Review sustainable approaches for T2DM reversal and update practitioners	Various bariatric and nonsurgical methods	Various lifestyle interventions	Highlights multiple methods for potential T2DM reversal but no standardization	Cohort study	Discusses evidence for diabetes reversal through various interventions including bariatric surgery and lifestyle changes
Chumakova-Orin et al., 2021 [34]	Obese patients (age and BMI not specified)	To assess T2DM outcomes following bariatric surgery and compare with the best medical management	Roux-en-Y gastric bypass and biliopancreatic diversion	Best medical management	Bariatric surgery yields better T2DM resolution than medical management alone	Cohort study	Higher rates of T2DM resolution in GBP and Biliopancreatic diversion compared to other procedures
Vetter et al., 2012 [39]	Adults with obesity (specific age/BMI not detailed)	Evaluate the efficacy of bariatric surgery on diabetes remission	Various bariatric surgeries (not specified)	None specified	45%-95% rate of diabetes remission, depending on procedure	Cross-sectional study	Greater weight loss correlated with higher diabetes remission rates; mechanisms include caloric restriction and altered gut hormone secretion
Yamaoka et al., 2019 [40]	20,113 participants; specific age/BMI not detailed	To compare the effectiveness of lifestyle modifications with other treatments in preventing T2DM	Lifestyle modifications (diet, exercise)	Standard treatments or placebo	OR of 0.46 (95% CI: 0.33 to 0.61) for LM vs. standard intervention; significant reduction in T2DM onset	Cross-sectional study	Lifestyle modification significantly reduces the onset of T2D compared to standard and placebo interventions. It is at least as effective as nine other treatments
Schauer et al., 2005 [35]	240 patients with T2DM (mean age 48 years, BMI 50.1 kg/m²)	To evaluate clinical parameters associated with diabetes improvement after LRYGBP	Laparoscopic Roux-en-Y gastric bypass (LRYGBP)	None	83% complete resolution of T2DM; significant reduction in diabetes medication use	Cohort study	Significant weight loss (60% excess weight); better outcomes in patients with shorter duration of diabetes. Early surgical intervention is recommended
Marchetti et al., 2014 [36]	252 patients (79 with T2DM, age not specified, BMI not specified)	Analyze the effect of RYGB on the control and treatment of obesity-related comorbidities	Roux-en-Y gastric Bypass	None specified	37.9% total T2DM remission; reduction in medication use for T2DM and hypertension	Retrospective cohort study	RYGB effective for T2D and hypertension remission; significant reduction in medication use among participants
Gill et al., 2010 [41]	673 patients with T2DM, mean BMI 47.4 kg/m² (range 31.0-53.5)	Systematically review the effect of LSG on T2DM	Laparoscopic sleeve gastrectomy (LSG)	None	66.2% resolved T2D, 26.9% improved, mean decrease in blood glucose of -88.2 mg/dL, HbA1c of -1.7%	Cross-sectional study	Most patients experienced resolution or improvement in T2D markers post-LSG, indicating LSG's potential as metabolic therapy
Lee et al., 2010 [52]	Moderately obese patients, aged 30-60 years	To determine the efficacies of two weight-reducing operations on diabetic control and the role of duodenum exclusion	Gastric bypass with duodenum exclusion (n = 30)	None	93% remission in gastric bypass group vs 47% in sleeve gastrectomy group (p = .02)	Double-blind RCT	Gastric bypass led to higher remission rates, greater weight loss, and better metabolic outcomes
Pournaras et al., 2011 [37]	34 obese T2DM patients undergoing bariatric surgery	Investigate T2DM remission rates postgastric bypass and banding; identify mechanisms of remission	Gastric bypass and gastric banding	Very low-calorie diet	72% remission in GBP vs. 17% in banding; HbA1c improved by 2.9% (GBP) and 1.9% (banding)	Prospective cohort study	Significant glycemic control improvement after GBP; rapid insulin resistance improvement in GBP; and enhanced GLP-1 responses may explain increased insulin production postsurgery
Brancatisano et al., 2008 [53]	Morbidly obese patients with T2DM, IGT, or metabolic syndrome (N = 682)	Assess the efficacy of the Swedish adjustable gastric band in T2DM, IGT, and metabolic syndrome	Swedish adjustable gastric banding	None reported	81% on oral hypoglycemic agents saw remission; HbA1c decreased from 8.0% to 6.1%; FBS decreased from 9.6 mmol/L to 5.7 mmol/L	Randomized controlled trial	38% excess weight loss at 12.5 months
Wallenius et al., 2018 [54]	33-64 years, BMI: LRYGB 42.1 ± 1.1, LSG 39.9 ± 1.2	Compare early weight-independent and later weight-dependent glycemic effects of LRYGB vs. LSG	Roux-en-Y gastric bypass (LRYGB) (n = 18)	None	Similar glycemic control; LSG showed higher peak insulin levels at two days postop	Randomized controlled trial	Similar glycemic control for both surgeries, but LRYGB had better BMI reduction and GLP-1 levels
Murphy et al., 2022 [55]	114 adults with T2DM, BMI 35-65 kg/m²	To determine if SR-LRYGB or LSG produces superior diabetes remission at five years	Silastic ring laparoscopic Roux-en-Y gastric bypass (SR-LRYGB)	Laparoscopic sleeve gastrectomy (LSG)	Remission in 47% (SR-LRYGB) vs. 33% (LSG) patients; HbA1c < 6% without glucose-lowering medications	Single-center, double-blind RCT	SR-LRYGB provided superior diabetes remission (adjusted OR 4.5, p = 0.009) and weight loss (absolute difference 10.7%, p < 0.001) compared to LSG; similar early and late complications
Panunzi et al., 2016 [56]	727 patients; mean BMI not specified	To identify predictors of diabetes remission and glycemic control after bariatric surgery	Gastric only (GO) and gastric plus diversion (GD)	Medical management (n = 312)	64% surgical group vs. 15% medical group achieved diabetes remission (p < 0.001)	Randomized controlled trial	GO yielded 60% remission; GD yielded 76% remission
Zhou & Zeng, 2023 [42]	544 participants (specific age not provided, BMI < 35 kg/m²)	To compare the effect of surgical and nonsurgical treatments on diabetes remission in patients with mild obesity	Various bariatric surgeries	Nonsurgical treatments	Bariatric surgery showed higher rates of diabetes remission (OR: 25.06, 95% CI: 9.58-65.54); significant reductions in HbA1c (MD: -1.44) and FPG (MD: -2.61)	Cross-sectional study	Bariatric surgery is more effective than non-surgical treatment for diabetes remission and glycemic control in T2D patients with BMI < 35 kg/m²; more effective in Asians
Huang et al., 2024 [43]	Not specified; focus on obesity-related conditions	Review the effectiveness of bariatric surgeries on obesity-related comorbidities	Laparoscopic Roux-en-Y gastric bypass (LRYGB), laparoscopic sleeve gastrectomy (LSG), loop duodenojejunal bypass with sleeve gastrectomy (LDJB-SG)	None specified	LRYGB shows higher remission rates for T2DM than LSG	Cross-sectional study	LRYGB has superior resolution rates for metabolic diseases compared to LSG; LDJB-SG is a new technique showing promise
Mirghani et al., 2023 [44]	Middle-aged participants (specific age and BMI not provided)	To assess the impact of bariatric surgery on weight loss and glycemic control in obesity and T2DM	Various bariatric procedures (not specified)	Medical management and lifestyle changes	Bariatric surgery consistently aids in weight reduction and glycemic control	Cross-sectional study	Significant benefits in weight loss and glycemic control; outcomes vary by surgery type and patient factors
Roebroek et al., 2024 [57]	Adolescents aged 14-16 years with severe obesity	Investigate the efficacy and safety of bariatric surgery in adolescents after insufficient weight loss from MLI	Laparoscopic adjustable gastric banding (n = 29)	Multidisciplinary lifestyle intervention (MLI) (n = 30)	The surgery group lost 11.2% of weight; the control group gained 1.7%; improved insulin and lipid profiles in the surgery group	Randomized controlled trial	Significant weight loss and improvements in glucose/lipid metabolism after surgery compared to MLI alone
Casella et al., 2015 [45]	Morbidly obese subjects (specific age and BMI not provided)	To assess changes in insulin sensitivity and secretion after sleeve gastrectomy	Sleeve gastrectomy	None reported	Improved insulin sensitivity; decreased insulin secretion; a significant increase in GLP-1 levels	Cross-sectional study	Insulin sensitivity improved; the major driver was GLP-1 secretion
Douros et al., 2019 [46]	Patients with morbid obesity and T2DM	To explore the effects of bariatric surgery on islet function, insulin secretion, and glucose metabolism	Roux-en-Y gastric bypass (RYGB), vertical sleeve gastrectomy (VSG), biliopancreatic diversion (BPD)	Intensive lifestyle/medical management	Surgery shown to be superior in improving diabetes	Cross-sectional study	Evidence supports that bariatric surgery improves glucose metabolism and insulin sensitivity, often before significant weight loss
Ji et al., 2021 [47]	Not specified	To review the effects of bariatric surgery on obesity and its comorbidities, mechanisms of improvement	Various forms of bariatric surgery	None specified	Improvement in obesity and comorbidities like T2DM and NAFLD; hormonal changes noted	Cross-sectional study	Bariatric surgery effectively decreases obesity and associated comorbidities; hormonal pathways are involved in improvements

The main interventions evaluated were diverse forms of bariatric surgery, notably RYGB and SG. These procedures are recognized for their possibility to induce significant weight reduction and metabolic alterations that can lead to enhanced diabetes administration. Some studies additionally investigated other surgical selections, like AGB and biliopancreatic diversion. In numerous cases, these surgical interventions were matched against standard nonsurgical therapies, like lifestyle modifications and pharmacological management, which offered a baseline for assessing the efficacy of surgical approaches.

The key outcomes determined in these studies included rates of diabetes remission, characterized as accomplishing regular glycemic levels without the necessity for diabetes medications, and glycemic regulation, often assessed through changes in hemoglobin A1c (HbA1c) levels. Numerous studies reported that patients who underwent bariatric surgery experienced significantly greater improvements in HbA1c compared to those who received nonsurgical interventions. Additionally, some studies examined the effects of surgery on other diabetes-related comorbidities, further emphasizing the broader health benefits associated with successful weight loss.

Findings of included studies in the systematic review

Efficacy of Bariatric Methods

RYGB provides an impactful solution for weight loss and diabetes management alike. Consider the findings from Schauer et al. [35] demonstrating average weight reductions of 30% to 40% of the total body weight following the procedure. More impressively, RYGB proved highly effective at promoting insulin sensitivity and driving down HbA1c levels. Approximately 40% of patients achieved T2DM remission within just one year postsurgery. After five years, as many as 80% saw their diabetes go into remission. The root mechanisms involve hormonal alterations stimulating weight loss while simultaneously enhancing glucose processing. SG offers a dual benefit of substantial weight loss coupled with dramatic decreases in HbA1c. As Mirghani et al. [44] revealed, the average HbA1c drop in the first two years postsurgery ranged from 1.5% to 3%. Much of this success stems from removing a sizable portion of the stomach and curbing ghrelin levels to induce a lasting sense of fullness. This aids in long-term weight management and metabolic health maintenance. Of the three bariatric procedures, AGB demonstrates the lowest rates of T2DM remission relative to RYGB and SG. According to the literature highlighted by Madsen et al. [31], only approximately 30% of patients using gastric bands achieve remission. This often tied to inadequate weight loss and inconsistent patient compliance with recommended dietary changes and follow-up appointments.

Sustainability of Remission

Roebroek et al. [57] presented long-term data in an extensive longitudinal study demonstrating that patients who underwent RYGB retained substantial weight reduction and metabolic control for a minimum of five years postoperation. Their findings revealed that nearly 70% of patients who attained diabetes remission at the one-year mark maintained it at the five-year point, underscoring the impact of sustained lifestyle changes in tandem with surgical intervention. Casella et al. [45] further reinforced the relativity of effectiveness between bariatric treatments in their comprehensive meta-analysis, highlighting SG patients exhibited anywhere from 60% to 70% probability of enduring remission for T2DM beyond five years, though results can fluctuate notably with adherence to individual postsurgical protocols. The analysis brought together a wealth of data evaluating the long-term impact of various weight loss surgeries, allowing for meaningful comparisons of outcomes over an extended timeframe.

Patient Characteristics Influencing Outcomes

As evidenced by Ji et al. [47], younger patients diagnosed with diabetes more recently often experience more successful metabolic outcomes after bariatric surgery compared to older patients with a longer history of battling the disease. Specifically, the study noted that individuals under 50 years old and those diagnosed within the last half decade demonstrated an enhanced likelihood of remission, implying value in earlier intervention. Research also underscores that the initial degree of excess weight portends a postoperative remission rate. In their study, Douros et al. [46] reported that those with BMIs over 40 at the time of procedure exhibited less frequent remission, highlighting how critical patient selection and customizing surgical approaches prove based on baseline weight and coexisting illnesses. The inverse relationship between preoperative adiposity and postbariatric glycemic control demands attentiveness to individuals' starting points on the scale.

Impact on Comorbidities

While Brancatisano et al. [53] reported significant improvements not only in T2DM but also in related conditions such as hypertension, dyslipidemia, and sleep apnea postbariatric surgery, their multifaceted investigation uncovered that patients experienced a profound 50% reduction in hypertension medication usage within merely two years, demonstrating the extensively transformative benefits of weight loss surgery on both physical and mental well-being.

Quality of Life Improvements

Marchetti et al. [36] emphasized patients' accounts of vastly enhanced quality of life and psychological prosperity following their procedure, as they cited noteworthy decreases in anxiousness and depressive symptomatology. The dramatic improvement in physical mobility and overall health led to much greater participation in social and recreational physical activities, further cultivating life satisfaction in multifarious ways.

Safety and Complications

Numerous operations were meticulously scrutinized concerning their security outlines by Yamaoka et al. [11] revealing that while difficulties like pollution, deficits, and weakening deficiencies can arise, they tend to seldom happen. Their examination indicates a 30-day complexity rate of about 3% to 5% for RYGB and gastric sleeve limited, which is kind when contrasted with the potential durable well-being advantages these medical procedures give. Grasping and controlling these dangers is pivotal both for patients and human service suppliers to guarantee educated basic leadership and postoperative achievement. Moreover, ensuring that well-being risks are comprehended requires an individualized technique, as each patient encounters abstract and actual difficulties distinctly throughout the time following careful intervention. In this manner, customized well-being correspondence is fundamental for patient success.

Postoperative Follow-Up and Support

Numerous studies collectively demonstrate the pronounced linkage between enduring success in diabetic remission and judiciously structured postoperative medical backing as well as assistance systems. Investigation led by Vetter et al. [39] signifies that continuous participation with health professionals, including nutritionists and psychologists, substantially enhances conformity to lifestyle transitions, guiding to more auspicious long-term outcomes.

Adherence to Dietary and Lifestyle Changes

Commitment to dietary directives is a pivotal determinant impacting long-term remission upshots. Research, like that conducted by Chumakova-Orin et al. [34], discloses that patients who actively take part in supportive groups and educational programs exhibit notably improved commitment to prescribed lifestyle variations, which relates to sustained weight reduction and diabetes management. While weight loss surgery offers a promising treatment for T2DM, further insights remain imperative. Varied impacts emerge from distinct procedures and patient traits, with remission sensitive to comorbidities. Lengthy successes necessitate thorough postoperative support. These nuanced outcomes illuminate efficacy's dependencies and underscore personalized care's importance for obesity and diabetes. No single method suits all; close monitoring improves understanding of each approach's durability. Continued efforts to unpack surgery's complex effects will better inform sensitive, supportive care for individuals.

Discussion

The efficacy of weight loss surgery and improving metabolic health in T2DM patients is well-established. Studies including Shibib et al. [33] and Wazir et al. [38] provide strong evidence that RYGB and SG result in high remission rates for diabetes, reaching as high as 80% and 60%, respectively. These procedures yield metabolic advantages beyond weight loss through altered gut hormone secretion and increased insulin sensitivity. In contrast, Chasapi et al. [58] highlighted the tendency for AGB to produce lower diabetes remission rates primarily because its mechanism relies solely on physical restriction rather than the metabolic effects seen with gastric bypass or SG. This disparity underscores the need to tailor surgical interventions to individual patient profiles, accounting for obesity severity and metabolic condition. Recent research by Dolin et al. [59] and He et al. [60] reinforce these findings by indicating that RYGB results in reduced HbA1c levels and higher sustained remission rates relative to gastric banding or SG alone. Notably, the timing of surgery is pivotal, as younger patients with newer diabetes diagnoses benefit dramatically more from metabolic surgery. This proposes that early intervention may be critical for maximizing long-term metabolic outcomes.

While the long-term efficacy of diabetes remission after bariatric surgery is crucial, sustaining results depends on complex factors. Casella et al. [45] found that approximately 70% of those achieving remission within the first year maintained it five years later, underscoring how lifestyle changes can cement impact. Their study highlights the necessity of engagement with adjustments postoperation, suggesting surgery alone provides incomplete benefits without perpetual commitment. However, Koliaki et al. [61] revealed variation in long-term remission rates depending on procedure, such as 60%-70% for SG. This variability emphasizes that adhering to aftercare plans plays a decisive role in sustained triumph. Taken together, these findings imply surgical achievement alone does not guarantee lasting recovery without ongoing support and personalized postsurgical strategies tailoring to every patient. Sustaining remission relies on both initial success and commitment to long-term wellness.

It is clear from prior investigations that eliminating excess weight and hyperglycemia sooner rather than later results in more beneficial results for patients. Ji et al. [47] demonstrated in their study that younger individuals and those newly diagnosed with T2DM tended to have more auspicious postoperative progress. This underscores the value of surgical intervention early on and underscores the potential for more advantageous metabolic consequences when addressing obesity and diabetes without delay. In contrast, Aminian et al. [62] report that higher preoperative BMI was inversely linked to remission rates, signifying that persons with more severe obesity may necessitate extra steps to optimize outcomes from surgery.

Recent research has provided valuable insight into the far-reaching benefits of bariatric surgery. Huang et al. [43] strikingly found that weight loss procedures reduced high blood pressure and abnormal cholesterol levels by 50%, implying tremendous potential for overall wellness beyond kilograms shed. However, Syn et al. [63] discovered psychosocial enhancements as well, such as upgraded mental health and quality of living, highlighting surgery's multifaceted impacts. While physiological advances are substantial, their findings point to psychological and social factors being equally pivotal. McGlone et al. [64] strengthened these conclusions, confirming that bariatric operations conduce to prolonged improvements in health-related quality of life. Most notable was that participating in support groups augmented psychological consequences, stressing the necessity of a holistic approach addressing both flesh and spirit in postoperative care plans. The studies cumulatively show that surgery meaningfully alleviates comorbid diseases and enhances well-being by targeting both the body and brain. A treatment addressing whole-person health yields benefits far surpassing a narrow focus on weight alone.

The safety profile of bariatric surgery, as outlined by Roebroek et al. [57], indicates that complications tend to transpire infrequently in spite of their potential occurrence. The reported complication rate of 3% to 5% for RYGB and SG is comparatively auspicious in relation to the long-term health benefits these surgeries offer. Nevertheless, complications involving nutritional insufficiencies underscore the necessity for individualized postoperative care catered to each patient. Recent technological enhancements in surgical techniques and perioperative care, as emphasized in the study by Alqunai et al. [65], have improved both the protection and effectiveness of these procedures, proposing that constant innovation in this field is principally important for optimizing patient outcomes. However, continuous progress is still required to further limit potential issues and validate the long-term sustainability of these benefits over various styles of patients and medical histories.

Limitations and recommendation of the study

There are important limitations, including the heterogeneity of included studies in terms of methodology, populations, and definitions of diabetes remission, making comparisons challenging and affecting overall conclusions. Furthermore, there is a risk that studies with subtly negative results are not published, and thus, the effectiveness of bariatric surgery may be overestimated. The heterogeneity of the study quality may dilute the findings from the review, and lack of long-term data inhibits assistance. Additionally, the use of group data instead of patient-specific outcomes can hide heterogeneity within subgroups. Higher quality RCTs, larger participant numbers using standardized protocols, and longer follow-ups are also needed to strengthen the evidence base for this approach. A greater understanding of the effect of bariatric surgery on T2DM remission will also result from the incorporation of patient-reported outcomes and confounding factors.

Clinical implication

It highlights bariatric surgery as a promising option for the treatment of T2DM, specifically in patients with obesity, which should encourage clinicians to consider not only its effects on body weight but also its use as a tool for achieving remission of diabetes. This underscores the importance of a multidisciplinary approach in which surgeons, endocrinologists, dietitians, and mental health professionals all work together to ensure thorough assessments prior to surgery and postoperative care. It is important that we follow-up on these patients long-term after this procedure to see if weight loss can be sustained, and it will cause rapid glycemic control, at least for some cases, so that they can be treated promptly. Moreover, counseling patients on the pros and cons of bariatric surgery can increase motivation for lifestyle changes as well as compliance with postoperative recommendations. This study may also contribute to the prioritization of public health policies by providing evidence for prioritized access to bariatric surgery as a crucial component in managing diabetes prevention programs. Finally, it has the potential to drive new research in understanding how we could optimize surgical approaches and examine the mechanisms of diabetes remission following bariatric surgery which can expand our evidence base for future guidelines.

## Conclusions

Bariatric surgery is a highly effective intervention for individuals with T2DM, particularly those with obesity. It leads to significant weight loss and improved glycemic control through mechanisms that reduce stomach size and alter hormonal responses. This surgery not only helps many patients achieve remission from diabetes but also decreases the risk of obesity-related health issues. Beyond physical health, patients often report enhanced psychological well-being and quality of life. Overall, bariatric surgery can transform the health trajectory of select individuals, offering them a renewed sense of control and improved overall health.
